# Malaria profiles and challenges in artemisinin resistance containment in Myanmar

**DOI:** 10.1186/s40249-017-0292-4

**Published:** 2017-04-25

**Authors:** Thet Wai Nwe, Tin Oo, Khin Thet Wai, Shuisen Zhou, Johan van Griensven, Palanivel Chinnakali, Safieh Shah, Aung Thi

**Affiliations:** 1 National Malaria Control Programme, Department of Public Health, Ministry of Health, Zabukyetthayay Road, Nay Pyi Taw, Myanmar; 2 Department of Medical Research, Ministry of Health, Nay Pyi Taw, Myanmar; 3 National Institute of Parasitic Diseases, Chinese Center for Disease Control and Prevention, Beijing, China; 4 Institute of Tropical Medicine, Antwerp, Belgium; 5 Jawaharlal Institute of Postgraduate Medical Education and Research (JIPMER), Puducherry, India; 6 Operational Research Unit (LuxOR), Médecins Sans Frontières – Operational Centre Brussels, Luxembourg City, Luxembourg

**Keywords:** Malaria, Artemisinin resistance, Myanmar

## Abstract

**Background:**

This study examined evolving malaria profiles from January, 2010 to December, 2014 to evaluate achievements and challenges of implementing measures to prevent and control spread of artemisinin resistance in Myanmar.

**Methods:**

Using National Malaria Control Programme (NMCP) data, a cross-sectional descriptive study of 52 townships in artemisinin-resistant containment areas in Myanmar was conducted. Annual program data were analysed, and trends over time are graphically presented.

**Results:**

In the 52 study townships populated by 8.7 million inhabitants, malaria incidence showed a decreasing trend from 10.54 per 1 000 population in 2010 to 2.53 in 2014, and malaria mortalities also decreased from 1.83 per 100 000 population in 2010 to 0.17 in 2014. The proportion of confirmed to total tested malaria cases also decreased from 6 to 1%, while identification of cases improved. All cases from all parasites species, including *Plasmodium falciparum*, decreased. Coverage of LLIN (long-lasting insecticidal net)/ITN (insecticide-treated mosquito nets) and indoor residual spraying (IRS) was high in targeted areas with at-risk persons, even though the total population was not covered. In addition to passive case detection (PCD), active case detection (ACD) was conducted in hard-to-reach areas and worksites where mobile migrant populations were present. ACD improved in most areas from 2012 to 2014, but continues to need to be strengthened.

**Conclusions:**

The findings provide useful data on the malaria situation in artemisinin-resistant initiative areas, which may be useful for the NMCP to meet its elimination goal. These profiles could contribute to better planning, implementation, and evaluation of intervention activities.

**Electronic supplementary material:**

The online version of this article (doi:10.1186/s40249-017-0292-4) contains supplementary material, which is available to authorized users.

## Multilingual abstracts

Please see Additional file 1 for translations of the abstract into five official working languages of the United Nations.

## Background

The global malaria situation has shown substantial improvement after massive deployment of preventive and curative tools, including artemisinin-based combination therapy (ACT). However, the emergence of artemisinin resistance in Southeast Asia is currently considered the greatest threat to regional and global malaria control and elimination. Artemisinin-resistant parasites were first detected in the vicinity of the Thai-Cambodian border, then subsequently in eastern Myanmar where it borders with Thailand [1]. In 2009–2010, Myanmar had reported suspected artemisinin resistance based on the results of therapeutic efficacy studies [2]. Spread of artemisinin resistance from Southeast Asia to the Indian subcontinent and Africa would have devastating consequences [3]. Myanmar occupies a key position in containment of artemisinin-resistant strains, as it has the largest malaria burden in this region and it geographically links Southeast Asia and the Indian subcontinent. Moreover, the artemisinin resistance risk areas are particularly challenging for malaria control, due to the high rates of migration at the border areas, remote forested and mountainous areas, and reliance on private health care providers.

As a response to these challenges, the Myanmar Artemisinin Resistance Containment (MARC) project was initiated in 2010–2011 and implemented in 2011 [4, 5]. The goals of the MARC project were to prevent or, at a minimum, significantly delay the spread of artemisinin-resistant parasites within Myanmar and beyond its borders, and to reduce transmission, morbidity and mortality of *Plasmodium falciparum* malaria/drug-resistant malaria [1]. MARC activities commenced in 21 townships in 2012, and expanded to 52 in 2014 and 72 in 2015 to avoid further spread beyond these areas. Key activities in MARC areas included increasing access to early diagnosis and effective treatment, improving community case management through community and private sector approaches, banning artemisinin-based monotherapy, reintroducing indoor residual spraying (IRS) combined with long-lasting insecticidal net (LLIN)/insecticide-treated mosquito nets (ITN), increasing use of LLIN/ITN to target 100% population coverage (anticipating two persons per net), strengthening malaria prevention and treatment for migrants at their work-sites, and setting up malaria screening points, operational research, periodic surveys, and applying multi-sectoral approaches. The need for strengthening routine surveillance systems and operational research was also emphasized [2].

Despite the vital role of artemisinin resistance containment projects to prevent national, regional, and global spread of artemisinin drug resistance, there have been few studies that evaluated the coverage of key containment activities and their impact on malaria indicators over time in the MARC areas. This information is particularly significant, as the MARC region has recently committed to moving from containment of artemisinin resistance to malaria elimination, following the launch of the Asia Pacific malaria elimination roadmap by World Health Organization (WHO) [4]. This would require all countries to make rapid progress, but Myanmar is particularly challenged, with 89% of all malaria cases in the Asia Pacific Region and the second highest burden of disease areas outside of Africa [6].

Success in eliminating malaria in Myanmar will require knowledge on how well the containment project has succeeded so far. It is currently unclear how many MARC areas have achieved sufficient reductions in malaria incidence to proceed to the pre-elimination phase. In this study, we report on 52 targeted areas with implementation of the key containment activities (malaria testing, coverage of LLIN/ITN and IRS, and active case detection), and their impact on key malaria indicators (incidence, mortality, and the proportion due to *Plasmodium falciparum* vs other species) from 2010 to 2014.

## Methods

### Study design

A cross-sectional descriptive study was carried out using annual records of National Malaria Control Programme (NMCP) data.

#### Setting

Myanmar is located in Southeast Asia, and borders the Republic of China to the north and northeast, Laos to the east, Thailand to the southeast, Bangladesh to the west, and India to the northwest. Myanmar is divided administratively into Nay Pyi Taw Council Territory and 14 states and regions. It consists of 74 districts, 330 townships, 398 towns, 3 065 wards, 13 619 village tracts, and 64 134 villages. The main geographical features of Myanmar are the delta region and the central plains surrounded by mountains. Myanmar has a tropical climate and a population of 51 486 253, with an urban: rural population ratio of 30∶70. It has an area of 676 577.2 square kilometres, and population density per square kilometer is 76.1 [7, 8].

Myanmar is one of the three countries in the WHO SEARO (Southeast Asia Region Office) Region that has the highest rates of malaria mortality and morbidity (Indonesia, Pakistan and Myanmar). Approximately 40 million people (72% of the total population) live in malaria risk areas, and 284 of the 330 townships in Myanmar are malaria endemic. A total of 205 658 confirmed malaria cases and 92 deaths were reported in 2014. Most malaria transmission occurs in forested foothill zones below an altitude of 1 000 m. High-risk groups are those residing near or in forests, worksites, projects and migrants [9, 10].

#### Study setting

According to the Global Plan on Artemisinin Resistance Containment, Myanmar was divided into three tiers. Tier 1 had evidence of artemisinin resistance, tier 2 had significant influx of inhabitants from tier 1 areas, especially those immediately bordering tier 1, and tier 3 was comprised of areas with no evidence of artemisinin resistance and limited contact with tier 1 areas [11]. NMCP, together with implementing partners, initiated immediate containment activity in July 2011, and the scaling-up control activities were conducted in tier 1 areas. The number of targeted townships was expanded to 52 townships in 2013, and among these, 48 were tier 1 and four were tier 2. To implement containment activities, NMCP and implementing partners were assigned project areas and tasks, and reporting to the NMCP was monthly by public sectors every three months by private non-government organizations (NGOs).

#### Study population

**Fig. 1:**
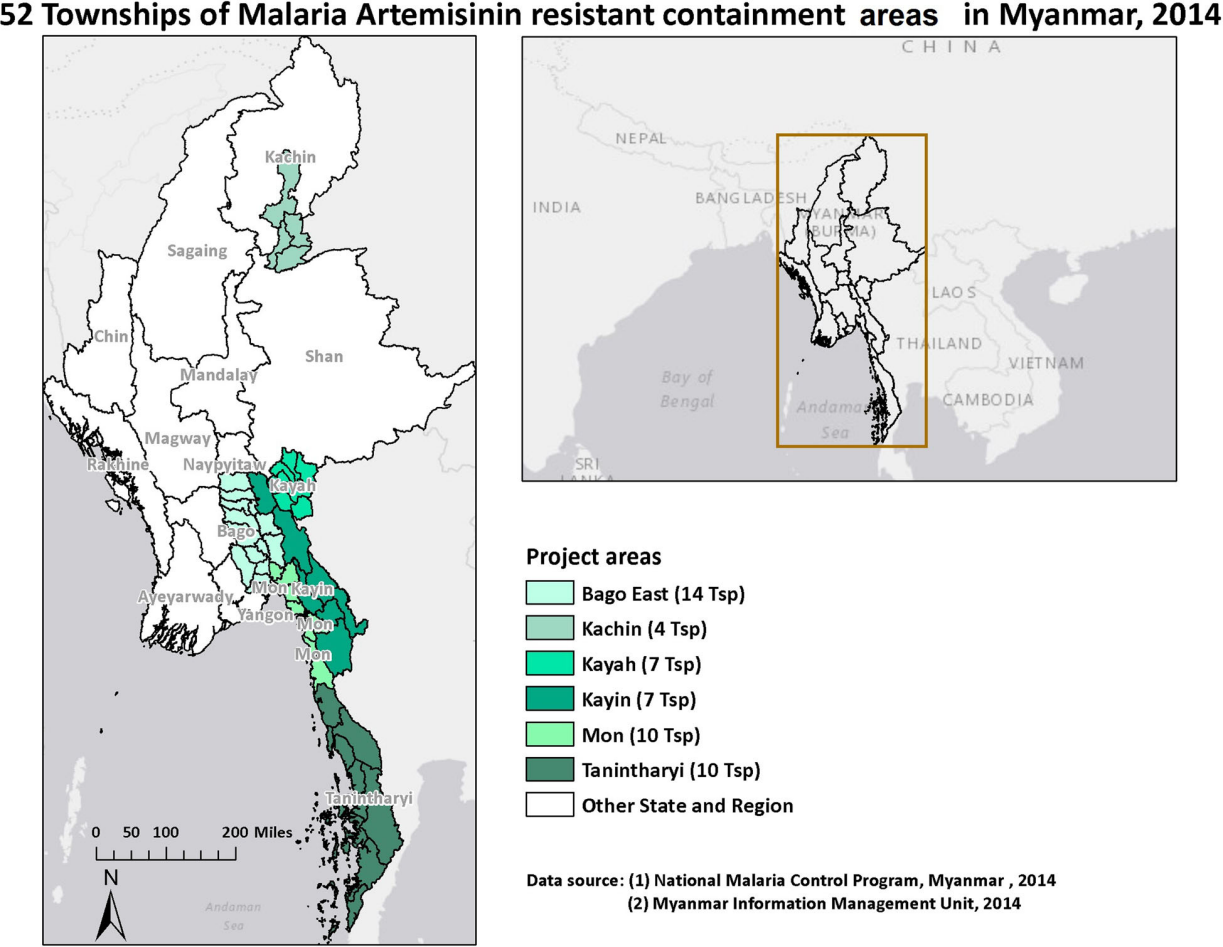
Study sites in the Artemisinin-resistant containment areas

The study was conducted in 52 townships of artemisinin-resistant containment areas in Myanmar that have a total of approximately 8.7 million inhabitants, and included 14 townships of the Bago East region, 7 townships in Kayah State, 7 townships in Kayin State, 10 townships in Mon State, 10 townships in the Tanintharyi region, and four townships in Kachin State (see Fig. 1).

#### Study duration

The study was conducted between July 2015 and March 2016, using NMCP data collected from years 2010 to 2014. Over this period, development partners changed from time to time, and depending on changing donors, project titles transitioned from national malaria prevention and control activities in 2010 to MARC activities during 2011 to 2103, and to Regional Artemisinin Resistance Initiative (RAI) activities in 2014.

#### Variables, data sources, and collection

The annual data of NMCP (2010–2014) for the 52 townships were obtained from their registers and records, and were double-checked using volunteer register data recorded by WHO Myanmar. NMCP data were recorded and compiled by state and regional vector-borne disease control programs, based on program registers, village health volunteer reports, and public health sector reports, including hospitals. Among the artemisinin-resistant containment areas in Myanmar, the 2010–2014 annual data of malaria cases, malaria deaths, population, confirmed malaria cases, tested malaria cases, *Plasmodium* species data, LLIN/ITN distribution data, IRS activities, and active case detection activities were collected and checked by double data entry.

### Analyses and statistics

NMCP data collected during 2010–2014 in the 52 townships selected for this study. After data cleaning and editing, all information was entered into Microsoft Excel and Epi data (version 3.1). We reviewed and analyzed data on malaria morbidity, mortality, and proportion of confirmed to tested cases; proportions of *P. falciparum*, *P. vivax* and other species; and coverage data of LLIN/ITN and IRS. Trends over time were graphically depicted.

Data are presented as: 1) malaria morbidity rate, the number of people with confirmed malaria per 1 000 population residing in the 52 townships during the reporting year (population data are based on the estimation of population growth rate); and 2) malaria mortality rate (number of deaths by confirmed malaria per 100 000) during the reporting year (data for deaths obtained from hospital-based data). Malaria cases were patients with malaria parasites confirmed by rapid diagnostic testing or microscopic examination. Tested cases were those screened and tested for malaria by various service providers. Malaria species identification of confirmed cases was mostly obtained from rapid diagnostic tests (RDT) and microscopic examination. Microscopically identified data were not available for all confirmed cases. Coverage data of LLIN and ITN are analyzed according to population covered by distributed LLINs within the total at-risk population.

### Ethics

Ethics approvals were obtained from the Ethics Review Committee of Department of Medical Research, Ministry of Health, Myanmar, and the Union Ethics Advisory Group (International Union against TB and Lung Disease).

## Results

Fifty-two townships in artemisinin resistance containment areas were categorized according to states and regions, with a total of six areas and five years of annual data. We examined impact and trends in malaria indicators and coverage and implementation of key activities.

### Impact on and trends in malaria indicators

#### Malaria incidence and mortality

**Fig. 2:**
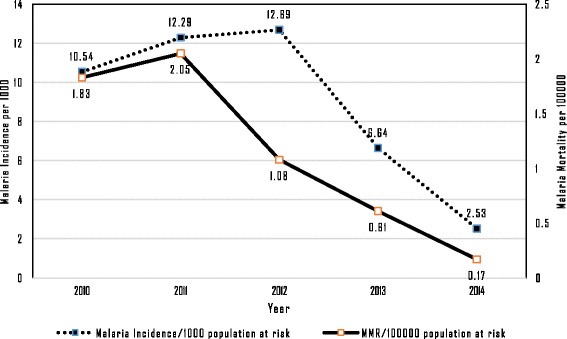
Trends of malaria incidence and malaria mortality rate within artemisinin-resistant containment areas, Myanmar, 2010–2014

**Fig. 3:**
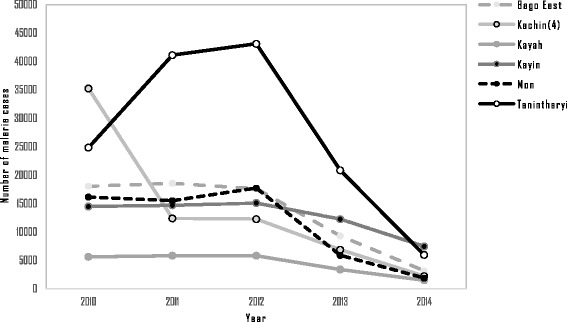
Malaria cases within artemisinin-resistant containment areas (states and regions), Myanmar, 2010–2014. Kachin (4): Only four townships of Kachin state

**Fig. 4:**
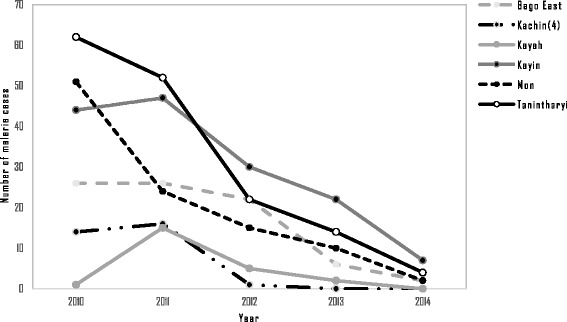
Malaria deaths within artemisinin-resistant containment areas (states and regions), Myanmar, 2010–2014. Kachin (4): Only four townships of Kachin state

Malaria incidence in the artemisinin-resistant containment areas decreased from 10.54 per 1 000 population in 2010 to 2.53 per 1 000 in 2014 (Fig. 2). By 2014, 15 of the 52 townships had reached the pre-elimination threshold (below 1/1 000) of annual parasite incidence (API). The malaria mortality rates also decreased from 1.83 per 100 000 in 2010 to 0.17 in 2014. The 52 townships were categorized into six states and regions and among these areas. Tanintharyi had the highest number of cases in 2012, but there were remarkable reductions in numbers of malaria cases after 2012 (see Fig. 3). The other states and regions showed steady decreases from 2010 to 2014, as shown in Fig. 3. Although Kachin state showed an abrupt decrease in malaria cases between 2010 and 2011, only four townships of this state were involved in artemisinin containment projects during our study period. Using hospital-based yearly reporting, state and regional malaria deaths from 2010 to 2014 are presented in Fig. 4. Within these areas, Tanintharyi had the highest mortalities in 2010 and 2011, then they decreased gradually. Kayin state had the highest mortality in 2014. The other states and regions showed a decreasing trend of malaria deaths from 2010 to 2014.

### Coverage and implementation of key containment activities

#### Malaria testing

**Fig. 5:**
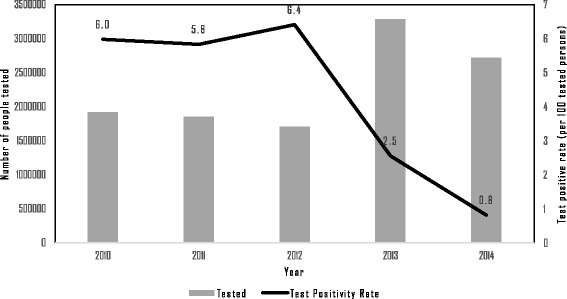
Number of people tested and Test positivity rate in artemisinin-resistant containment areas, Myanmar, 2010–2014

**Fig. 6:**
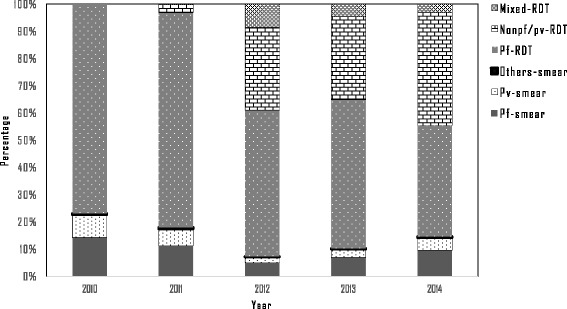
Proportion of malaria caused by *Plasmodium falciparum*, *P. vivax* and other species within artemisinin-resistant containment areas, Myanmar, 2010–2014. Pf-smear- *Plasmodium falciparum* cases confirmed by microscopically, Pv-smear- *Plasmodium viva* cases confirmed by microscopically, Other-smear- Other *Plasmodium* cases confirmed by microscopically, Pf-RDT- *Plasmodium falciparum* cases confirmed by rapid diagnostic test, Nonpf/pv-RDT- Other *Plasmodium* cases confirmed by rapid diagnostic test, Mixed-RDT- Mixed *Plasmodium* cases confirmed by rapid diagnostic test

**Fig. 7:**
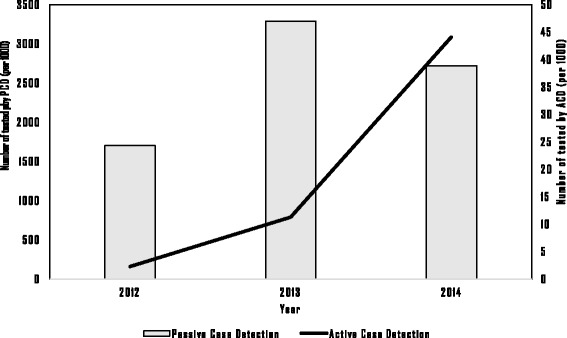
Number of tested persons by ACD and PCD within artemisinin-resistant containment areas, Myanmar, 2012–2014. ACD-Active Case Detection conducted in hard -to-reach areas and worksites of mobile/migrant population to fill the gap of PCD. PCD-Passive Case Detection, all tested cases in all health facilities. Kachin (4)- only four townships of Kachin state

Figure 5 shows annual numbers of people tested for malaria and confirmation by microscope or RDT. The numbers of people tested increased from 1.9 million in 2010 to 2.7 million in 2014, but the increase was only observed beginning in 2013, while a decrease in the proportion of confirmed cases among those tested was observed. The test positivity rate decreased from 6% in 2010 to 0.8% in 2014. Figure 6 shows the numbers of malaria mosquito species identified each year by RDT or microscopically. Since most of the registered malaria cases were confirmed by RDT, these comprise the highest proportion. As shown in Fig. 6, the *Plasmodium falciparum* species detected by RDT had a decreasing trend. The *Plasmodium* species ratio within study sites was evaluated for 2014, and *Plasmodium falciparum*, *Plasmodium vivax* and other species had a 65∶32∶3 ratio. Active case detection (ACD) activities in artemisinin-resistant areas during 2012–2014 are compared to passive case detection (PCD) in Fig. 7. In addition to PCD, ACD was conducted in hard-to-reach areas and worksites where mobile migrant populations were present. ACD improved in most areas from 2012 to 2014.

#### Coverage of ITN, LLIN and IRS activities

**Fig. 8:**
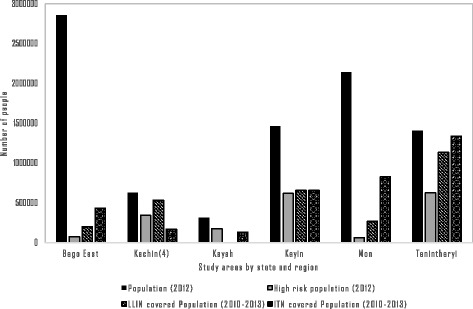
LLIN/ITN coverage in artemisinin-resistant containment areas, Myanmar, 2010–2013. LLIN- Long Lasting Insecticidal Net, ITN- Insecticide Treated Net, Kachin (4)-only for four townships in Kachin state, *Tanintharyi region’s ITN combination data (2010–2014) was more than total population because of repeated activities and so imputed to be equal to total population of Tanintharyi in this figure. 2012 data is used because there was available data for risk area stratification of all states and regions

LLIN and ITN coverage in study areas by states and regions during 2010–2013 are shown in Fig. 8. Coverage data of LLIN and ITN are analyzed according to population covered by distributed LLINs within the total at-risk population. As LLIN distribution and ITN activities were prioritized to high-risk areas, those populations in 2012 are also included in the Figure. The Tanintharyi region had the highest coverage for both LLIN and ITN, and the Bago East region and Mon state had high coverage for risk populations over the four-year period, whereas Kayah state had relatively low coverage of ITN activities. Table 1 depicts IRS activities conducted in the study areas from 2010 to 2014. Implementation sites included 112 villages or camps or project sites of 8 townships during the study period. Most commonly used insecticides were DDT (75%) and Fendona, and few households refused IRS (see Table 1).

**Table 1:** Indoor residual spraying (IRS) activities within artemisinin-resistant containment areas, Myanmar, 2010–2014

Year	Townships	No of villages/sites	Covered household/building	Number of refusing homes	Insecticide amount (kg)
2010	Bago	2	36	0	190
	Phyu	2	2073	0	710
	Loikaw	1	80	0	10
	Bawlakhe	2	262	0	18
	Meset	2	195	2	79
2011	Bawlakhe	1	69	0	3
	Loikaw	4	262	0	18
2012	ShweKyin	14	3974	0	243
	ThanPhyuZayet	23	2838	0	150
	Kawthaung	13	8742	0	75
2013	Loikaw	2	259	0	50
2014	ShweKyin	27	4435	0	300
	ThanPhyuZayet	19	2149	94	83.3

## Discussion

In this population-based cross-sectional study from 2010 to 2014, we evaluated malaria indicators such as incidence, mortality rates, and proportion of confirmed cases over time to obtain malaria profiles of artemisinin-resistant containment areas in Myanmar. Other important impact indicators, such as malaria species proportion and coverage of ITN, LLIN and IRS, are also described. Within our study areas, we identified 15 townships reaching pre-elimination threshold, annual parasite incidence (<1/1 000 population). Mu et al. [12] report that there was a sharp decline in reported malaria incidence and mortality during 2015–2014, which reflects increased political and financial commitment of Myanmar. Moreover, training and deployment of over 40 000 community health volunteers from high-risk areas were keys to intervention, improved access to early diagnosis, and prompt treatment.

Our findings of rapid declines in malaria incidence and mortality within these study areas were achieved by implementations of the national malaria control program and collaborating partners. Various projects were implemented to address the spread of artemisinin resistance during the study period (2010–2014), including NMPC control activities, MARC (2011–2013), and RAI (Regional Artemisinin Resistance Initiative) (2014–2016). MARC was done in collaboration with the Myanmar Ministry of Health and its partners between July 2011 and 2013. From 2014 on, Myanmar became the main recipient of the GMS regional artemisinin resistance initiative (RAI) with GFATM (the Global Fund to Fight AIDS, Tuberculosis and Malaria) and Bill and Melinda Gates Foundation (BMGF) funding, and Three Disease Fund will continue to fund MARC, possibly until 2016 [5]. Although we analysed the reported data of the activities that occurred under the different projects over the study period, it is possible to describe epidemiological changes and impacts of programs.

In our study, a decreasing trend in malaria incidence and mortality was seen in all areas from 2010 to 2014. However, there are some inconsistent findings that indicate challenges in the implementation of program activities, such as changing funding sources and possible alterations in ecological and epidemiological situations. The increasing trend of malaria incidence from 2010 to 2011 reflects the implementation of MARC activities in 2011, specifically due to increased testing, which led to increased case finding and reporting. The NMCP annual report of 2013 stated that the changes were due to climatic and ecological changes, population migration (i.e., migrants who seek economic opportunities in rural economic frontier areas), economic development activities (such as forestry, mining, plantations and road-building), and development of the multi-drug resistant *P. falciparum* parasite [12, 13].

According Kyaw et al. (2014), MARC achieved improved access to quality diagnosis and treatment, increased coverage of personal protection (LLINs and IRS), and compliance with national drug policy [5]. Increased numbers were tested during study period. Testing facilities are provided by both passive and active case detection. ACD by health workers was conducted in communities and households in the hard-to-reach areas and worksites where mobile migrant populations are present. ACD is required to reach the uncovered areas of PCD by which suspected cases were tested in all health facilities and health posts (health centers), but it remained challenging. Not all planned areas could be covered during the study period because of its delayed commencement.

Moreover, we could not get all microscopically identified data for all confirmed malaria cases in the study although we had some data, which indicates the need for improved microscopic examination facilities. According to the available microscopic data, we observed a decreasing trend in the *Plasmodium* species in the areas where activities were implemented. Our findings indicate the need for enhanced malaria diagnostic facilities so that microscopic slide positivity rates for most cases can be reported. The WHO operational manual recommends that the microscopic slide positivity rate be less than 5% as disease surveillance for malaria elimination [14].

The Tanintharyi region had the highest malaria morbidity in 2011 (36.9 per 1 000 population), and with the implementation of the MARC Project, accomplished highest LLIN coverage. After high coverage of LLIN/ITN in 2012, there were decreasing trends of malaria morbidity and mortality rates in the Tanintharyi region. An informal consultation report on MARC mentioned that populations covered by LLIN distribution in targeted areas were calculated as one LLIN/ITN for two persons [15]. The distribution plan was based on area stratification defined as 1a = high risk, 1b = moderate risk, 1c = low risk, 2 = potentially risky, and 3 = without malaria [16, 17]. The other states and regions also had good coverage for high-risk areas. However, a slight increase in malaria deaths in 2014 was seen in Kayah state, where few LLIN and ITN activities occurred during most of the study years. LLIN/ITN coverage data impacted upon malaria morbidity and mortality, and therefore indicates the effectiveness of mosquito net distribution. We also reported on ITN activities in the study because LLIN cannot cover all the populations at risk and people still prefer to use ordinary nets. Mu TT [12] noted that distribution of insecticide-treated nets and increased IRS activities are a potential role for community health volunteers.

IRS activities were implemented in each state and region, but more comprehensive linkage between selection of epidemiologic sites and effectiveness of activities requires further elucidation. The operation manual of IRS indicates focus on epidemiologic hotspots that have significantly higher confirmed malaria cases or transmission activity compared to surrounding areas [18, 19]. The National Strategic Plan (2010–2015) mentioned that the effectiveness of IRS activities could be evaluated in combination with LLIN distribution coverage, since IRS in combination with ITN/LLIN is recommended in artemisinin-resistant areas to maximize protection of at-risk populations [20].

Data on migrants and mobile populations were not included in this study, as that information was not readily available. The authors of a report on mobility dynamics of migrant workers [21] stated that migrants have difficulty in accessing malaria-related information and malaria care providers. According to Kyaw et al. (2014), challenges faced by MARC are civil unrest in some areas, communication barriers in some areas, and geographically hard-to-reach areas that might be limiting coverage to targeted mobile/migrant populations [5]. Hence, more data collection from migrants and ACD are necessary. A conference at which the Myanmar government and non-government representatives met in Washington, DC to discuss shared efforts to eliminate malaria (2015) [22] stated that success in eliminating malaria in Myanmar relies on reaching all people in Myanmar, with effective and targeted deployment of malaria prevention and surveillance tools, along with expedited, quality diagnosis and treatment of those with malaria. The discussion from this conference recommends working together across political and cultural lines for ethnic, migratory, military, and border and other hard-to-reach populations. Mu et al. [12] presents malaria incidence in Myanmar (2005–2014) as a steady but fragile progress towards elimination.

Our study findings present malaria profiles in selected study areas and how artemisinin-resistant containment activities impacted upon the profile of malaria in Myanmar, and what activities should be strengthened. In a report about malaria in Asia [23], the authors stated that there is a multitude of challenges for combatting malaria. Our study findings suggest that reaching migrant populations, improving comprehensive microscopic examination facilities, and data recording and reporting systems are the main challenges for NMCP activities. Controlling the spread of artemisinin-resistant malaria is the key to eradicating malaria [24], and our findings indicate the areas that should be emphasized for more effective implementation and better planning when moving toward the goal of elimination.

There were also certain limitations to the study. Our study used information recorded by NMCP, as some data from other implementation partners (i.e., INGOs/NGOs) were not available to us; therefore, this might be a form of bias in assessing and interpreting the trends in this study. Data on some confirmed cases and malaria deaths could not be analyzed due to township-based differentiation. Hence, we needed to combine data of states and regions. Probable malaria cases that had been clinically diagnosed in 2010 and 2011 were not included in this study in order to increase specificity in analyzing data in this study. Although the accuracy of the data for all of these areas should be cautiously interpreted, they represent the majority of reported data for the study periods.

## Conclusions

The malaria profiles of 52 townships in artemisinin-resistant areas in Myanmar revealed the importance of implementing a comprehensive program. The study yielded useful data about artemisinin resistance containment activities that NMCP has been implementing, which may be applicable to planning and evaluating program activities. Decreasing trends of morbidity and mortality, increased coverage of LLIN/ITN, and increases in testing showed the strengths of malaria prevention and control activities of the national program. However, developing the data reporting system into an electronically registered system and encouraging implementing partners (INGO/NGOs) to coordinate with that reporting system for accurate appending or merging of data would be necessary. The resulting information from this study evaluates various aspects of malaria control activities in Myanmar and suggests ways to maintain current successes and reduce challenges for the planned pre-elimination for malaria in Myanmar.

## Additional file

Additional file 1: Multilingual abstracts in the five official working languages of the United Nations. (PDF 756 kb)

## Abbreviations

3DF: Three disease fund
ACD: Active case detection
Artemisinin resistance: by *Plasmodium falciparum*
Artemisinin: Antimalarial drug: Artemisinin
BMGF: Bill and Melinda Gates foundation
GFATM: The global fund to fight Aids: Tuberculosis and Malaria
GMS: Greater Mekong subregion
INGO: International non-governmental organization
IRS: Indoor residual spraying
ITN: Insecticide-treated mosquito nets
LLIN: Long-lasting insecticidal net
MARC: Myanmar artemisinin resistance containment project
NGO: Non-governmental organization
NMCP: National malaria control program
PCD: Passive case detection
RAI: Regional artemisinin resistance initiative
RDT: Rapid diagnostic test for malaria
SEARO: South East Asia regional office
WHO: World Health Organization

## References

[1] National Malaria Control Program (2013). Annual report on vector borne diseases control 2012.

[2] World Health Organization Country Office for Myanmar. Fact sheet for regional artemisinin initiative. 2014. http://www.searo.who.int/myanmar. Accessed 15 Aug 2015.

[3] Department of Health and World Health Organization. Strategic framework for Artemisinin containment in Myanmar (MARC) 2011–2015. World Health Organization, South-East Asia Regional Office; 2011.

[4] National Malaria Control Program (2014). Proceedings of annual review meeting 2013.

[5] Kyaw TT, Hlaing T, Thimasarn K, Mon KM, Galappaththy GNL, Plasai V, Ortega L (2014). Containing artemisinin resistance of plasmodium falciparum in Myanmar: achievements, challenges and the way forward. WHO South-East Asia J Public Health.

[6] GBC Health (2014). Business leaders and health experts gather in Yangon for first regional meeting on corporate sector engagement in malaria control in the Asia Pacific.

[7] Department of Health and Unicef. Health in Myanmar 2014. Republic of the Union of Myanmar: Ministry of Health; 2015.

[8] The 2014 Myanmar population and housing census. Department of Population, Ministry of Immigration and Population. 2015. http://www.dop.gov.mm. Accessed 9 Jul 2015.

[9] National Malaria Control Program (2015). Proceedings of annual review meeting 2014.

[10] WHO (2015). World malaria report 2014.

[11] WHO (2013). Emergency response to artemisinin resistance in the greater Mekong subregion: Regional frame work for action, 2013–2015.

[12] Mu TT, Sein AA, Kyi TT, Min M, Aung NM, Anstey NM, Kyaw MP, Soe C, Kyi MM, Hanson J (2016). Malaria incidence in Myanmar 2005–2014: steady but fragile progress towards elimination. Malar J.

[13] National Malaria Control Program (2015). Annual report on vector borne diseases control 2013.

[14] WHO (2012). Disease surveillance for malaria elimination: an operational manual.

[15] Thimasarn K (2011). To organize the informal consultation on Myanmar artemisinin resistance containment (MARC) framework during 4–5 April 2011 in Nay Pyi Taw.

[16] de Beyl CZ, Ohnmar, Maung TM, Meek S, Thimasarn K, Soe AY, et al. Myanmar Artemisinin Resistance Containment (MARC) survey: malaria awareness and prevention, Malaria Consortium, poster 1335, 2012. http://www.malariaconsortium.org/media-downloads/233. Accessed 15 Apr 2016.

[17] Evaluation of malaria risk micro-stratification strategy. Department of Health, UNICEF: Myanmar. 2010.

[18] WHO (2013). Indoor residual spraying: an operational manual for indoor residual spraying for malaria transmission control and elimination.

[19] Guidance for focal indoor residual spraying. President Malaria Initiative. 2015. https://www.pmi.gov/how-we-work/technical-areas/indoor-residual-spraying. Accessed 15 Apr 2016.

[20] National Strategic Plan (2012). Malaria prevention and control (2010–2015).

[21] Hlaing T, Wai KT, Oo T, Sint N, Min T, Myar S (2015). Mobility dynamics of migrant workers and their socio-behavioral parameters related to malaria in Tier II, Artemisinin Resistance Containment Zone, Myanmar. BMC Public Health.

[22] Working Together to Eliminate Malaria in Myanmar August 3, 2015 in Washington, D.C., USA, 2015. https://csis-prod.s3.amazonaws.com/s3fs- public/legacy_files/files/attachments/150804_Myanmar_Conference_Statement.pdf. Accessed 21 Nov 2015.

[23] Bhatia R, Rastogi RM, Ortega L (2013). Malaria successes and challenges in Asia. J Vector Borne Dis.

[24] The Three Millennium Development Goal Fund. Annual Report (January to December 2014). 2014. http://www.3mdg.org/en/publications/reports. Accessed 15 Apr 2016.

