# Supplementary material for: Malaria profiles and challenges in artemisinin resistance containment in Myanmar
Source: Infect Dis Poverty. 2017 Apr 25;6:76. doi: 10.1186/s40249-017-0292-4 (PMC5404679; doi:10.1186/s40249-017-0292-4)
Supplement: Additional file 1: — Multilingual abstracts in the five official working languages of the United Nations. (PDF 756 kb) [file 40249_2017_292_MOESM1_ESM.pdf]

Translation of the abstract into the six official working languages of the United Nations

مرتسم الملاريا والتحديات في احتواء مقاومة الأرتيميسينين في ميانمار

ثيت واي نيو، تين، خين ثيت واي، شيوسن تشو، يوهان فان جرينزفن، بالانيفل تشيناكالي، صافية شاه، أونج ثي

ملخص

خلفية: بحثت هذه الدراسة تطور مرتسمات الملاريا خلال الفترة من يناير 2010 إلى ديسمبر 2014 لتقييم الإنجازات والتحديات التي تواجه تنفيذ التدابير الرامية إلى منع ومكافحة انتشار مقاومة الأرتيميسينين في ميانمار.

الطرق: استخدام بيانات البرنامج الوطني لمكافحة الملاريا، أجريت دراسة وصفية مقطعية لـ52 بلدة في المناطق التي يوجد بها المقاومة للأدوية في ميانمار. وقد تم تحليل بيانات البرنامج السنوية، وعرضت الاتجاهات الزمنية بيانيا.

النتائج: في البلدات الـ52 التي شملتها الدراسة يسكن 8.7 مليون نسمة، ظهر أن حالات الملاريا تقلصت من 10.54 لكل 1000 نسمة في 2،010 إلى2،53 في عام 2014، وانخفضت وفيات الملاريا أيضا من 1.83 لكل 100000 نسمة في 2،010إلى 0،17 في عام 2014. والنسب المؤكدة من مجموع حالات الإصابة بالملاريا انخفضت أيضا من 6٪ إلى 1٪، في حين تحسنت القدرة على اكتشاف الحالات. قلت جميع الحالات من جميع أنواع الطفيليات، بما في ذلك المتصورة المنجلية. كانت تغطية الناموسيات المعالجة بمبيدات الحشرات ذات الأثر الطويل الأجل / الناموسيات المعالجة بمبيدات الحشرات والرش الثمالي داخل المباني عالية في المناطق المستهدفة مع الأشخاص المعرضين للخطر، حتى لو لم يتم تغطية مجموع السكان. بالإضافة إلى الكشف عن الحالات السلبية، أجري الكشف عن الحالات المصابة في مناطق ومواقع العمل التي يصعب الوصول إليها حيث تواجد السكان المهاجرين المنتقلين. تحسن الكشف عن الحالات المصابة في معظم المناطق 2012-2014، لكنه ما زال بحاجة إلى تعزيزه.

الاستنتاجات: تقدم النتائج بيانات مفيدة عن وضع الملاريا في مبادرة المناطق المقاومة للأدوية، والتي قد تكون مفيدة للبرنامج الوطني لمكافحة الملاريا لتحقيق هدف القضاء عليه. هذه الملامح يمكن أن تسهم في تحسين التخطيط والتنفيذ وتقييم أنشطة التدخل.

Translated from English version into Arabic by Mahmoud Sami, through

TRANSLATORS
WITHOUT BORDERS

缅甸疟疾疫情概况及其遏制青蒿素耐药性面临的挑战

Thet Wai New, Tin Oo, Khin Thet Wai, Shuisen Zhou, Johan van Griensven, Palanivel Chinnakali, Safieh Shah, Aung Thi

摘要

引言：本研究通过审查 2010 年 1 月到 2014 年 12 月的疟疾疫情，以评估缅甸在防控青蒿素抗药性传播措施所取得的成就和面临的挑战。

方法：利用缅甸国家疟疾防控项目（NMCP）的数据，对缅甸 52 个青蒿素抗性控制乡镇进行横断面描述性研究。分析每年疟疾项目的数据，并以图示其随时间变化的趋势。

结果：52 个研究乡镇中，人口共计 870 万，疟疾发病率从 2010 年的 10.54/千降至 2014 年的 2.53/千，死亡率也从 2010 年的 1.83/10 万降至 2014 年的 0.17/10 万。确诊病例数占检测总病例数的比例也从 6%降至 1%，同时确诊水平得到提升。各种疟疾（包括恶性疟）病例数均减少。尽管长效药物蚊帐（LLIN）、杀虫剂浸泡蚊帐和室内滞留喷洒尚未覆盖全部人群，但是在目标区域风险人群中的覆盖率较高。此外，除被动病例侦查之外，在难

以达到的地区和有流动人口的工作区开展主动病例侦查。从 2012 年到 2014 年，大多数地区的主动病例侦查工作逐年改善，但仍需加强。
结论：本研究为青蒿素抗性起源地区的疟疾状况提供了有用的数据，这有助于疟疾控制项目实现疟疾消除目标。这些资料有助于进一步优化干预措施的制定、实施以及评估。

Translated from English version into Chinese by Xin-Yu Feng, edited by Pin Yang

CHINESE CENTER FOR DISEASE CONTROL AND PREVENTION
NATIONAL INSTITUTE OF PARASITIC DISEASES

Profils du paludisme et défis à relever pour la maîtrise de la résistance à l'artémisinine au Myanmar

Thet Wai New, Tin Oo, Khin Thet Wai, Shuisen Zhou, Johan vanGriensven, Palanivel Chinnakali, Safieh Shah, Aung Thi

Résumé
Contexte: Cette étude porte sur l'évolution des profils du paludisme de janvier 2010 à décembre 2014, afin d'évaluer les accomplissements et des défis de la mise en œuvre de mesures visant à empêcher et à tenir sous contrôle la propagation de parasites du paludisme résistant à l'artémisinine au Myanmar.
Méthodes: Une étude transversale et descriptive de 52 agglomérations dans des zones de lutte contre la propagation de parasites résistant à l'artémisinine au Myanmar à été conduite en utilisant des données du programme national de lutte antipaludique (National Malaria Control Programme - NMCP). Les données annuelles du programme ont été analysées, et les tendances dans le temps ont été présentées de façon graphique.
Résultats: Dans les 52 agglomérations objets de l'étude comptant 8,7 millions habitants, l'incidence du paludisme a présenté une tendance à la baisse allant de 10,54 pour 1000 habitants en 2010 à 2,53 en 2014. Le taux de mortalité imputable au paludisme a également baissé de 1,83 pour 100 000 habitants en 2010 à 0,17 en 2014. La proportion de cas confirmés de paludisme a également diminué, passant de 6% à 1%. En outre, l'identification des cas a connu une amélioration. Tous les cas de paludisme, quelque soit le parasite originaire, y compris le *Plasmodium falciparum*, ont diminué. La couverture MILD (moustiquaires imprégnées d'insecticide longue durée) / MTI (moustiquaires traitées à l'insecticide) et la pulvérisation intradomiciliaire à effet rémanent (IRS) ont été très utilisées dans les zones visées avec des personnes à risque, bien que la totalité de la population n'ait pas été pas couverte. En complément à une détection passive de cas, une détection active de cas a également été menée à bien dans des lieux difficilement accessibles et des chantiers où des populations nomades et migrantes étaient présentes. La détection active de cas a connu une amélioration dans la plupart des zones de 2012 à 2014, mais doit encore être renforcée.
Conclusion: Les résultats de cette étude fournissent des données utiles sur la situation épidémiologique du paludisme dans des zones résistantes à l'artémisinine qui peuvent s'avérer essentielles pour que le NMCP atteigne son objectif d'éliminer le paludisme. Ces profils pourraient contribuer à une amélioration de la planification, de la mise en œuvre et de l'évaluation des activités d'intervention.

Translated from English version into French by Carole Salas, through

TRANSLATORS
WITHOUT BORDERS

Досье по малярии и вызовы, связанные с мерами по сдерживанию резистентности к артемизинину в Мьянме

Thet Wai New, Tin Oo, Khin Thet Wai, Shuisen Zhou, Johan van Griensven, Palanivel Chinnakali, Safieh Shah, Aung Thi

Аннотация

Базовая проблематика. В данном исследовании рассмотрены досье развития штаммов малярии за период с января 2010 года по декабрь 2014 года с целью оценки достижений и вызовов, связанных с осуществлением мер по предупреждению и борьбе с распространением резистентности к артемизинину в Мьянме.

Методы. При использовании данных Национальной программы борьбы с малярией (НПМ) проведено перекрестное описательное исследование в 52 населенных пунктах, относящихся к районам сдерживания распространения резистентности к артемизинину в Мьянме. Проанализированы данные годовой программы и показаны в графическом виде временные тенденции.

Результаты. В 52 исследуемых пунктах с населением 8,7 миллиона жителей, показатель заболеваемости малярией имел тенденцию к снижению: с 10,54 на 1000 человек в 2010 году до 2,53 — в 2014 году; показатель смертности от малярии снизился: с 1,83 на 100 000 человек в 2010 году до 0,17 — в 2014 году. Соотношение подтвержденных случаев к общему количеству случаев заболевания малярией также уменьшилось с 6% до 1% на фоне улучшения выявления случаев заболеваемости. Уменьшился показатель всех случаев заболеваемости всеми формами малярии, в том числе *Plasmodium falciparum*. В целевых районах высокого эпидемиологического риска отмечен высокий уровень охвата населения мероприятиями по распределению СОИДД (сетки, обработанными инсектицидом длительного действия) / ОИС (обработанные инсектицидами противомоскитные сетки) и мероприятиями по опрыскиванию внутренних помещений (ОВП), однако всеобщих охват так и не был достигнут. В труднодоступных районах и местах работ, которые по большей части представлены мигрантами и мобильными группами населения, помимо пассивного выявления случаев заболевания (PCD), проводилось и активное выявление случаев заболевания (ACD). В большинстве районов за период с 2012 по 2014 год показатель ACD улучшился, но и он требует дальнейших улучшений.

Выводы. Полученные результаты представляют собой полезные данные о ситуации по малярии в районах возникновения артемизининовой резистентности. Кроме того, результаты могут быть полезны НПМ в рамках достижения цели по ликвидации заболевания. Эти досье могут способствовать более эффективному планированию, имплементации и оценке интервенционных мероприятий.

Translated from English version into Russian by Anna Romanenko, through

TRANSLATORS WITHOUT BORDERS

Perfiles y desafíos que plantea la malaria en la contención de la resistencia a la artemisinina en Myanmar

Thet Wai New, Tin Oo, Khin Thet Wai, Shuisen Zhou, Johan van Griensven, Palanivel Chinnakali, Safieh Shah, Aung Thi

Resumen

Antecedentes: en el presente estudio se examinaron los perfiles evolutivos de la malaria durante el período comprendido entre enero de 2010 y diciembre de 2014 con el objeto de evaluar los logros y dificultades de la aplicación de medidas para prevenir y controlar la propagación de la resistencia a la artemisinina en Myanmar.

Métodos: valiéndose de los datos del Programa Nacional de Control de la Malaria, se llevó a cabo un estudio descriptivo transversal en 52 municipios en las zonas de contención de la resistencia a la artemisinina en Myanmar. Se analizaron los datos anuales del programa, y se presentaron las tendencias a lo largo del tiempo mediante gráficos.

Resultados: en los 52 municipios sometidos a examen, que cuentan con una población de 8,7 millones de habitantes, la incidencia de la malaria mostró una tendencia decreciente que pasó de 10,54 por 1.000 habitantes en 2010 a 2,53 en 2014, y la mortalidad a causa de la malaria también disminuyó del 1,83 por 100.000 habitantes en 2010 a 0,17 en 2014. Asimismo, la proporción total de casos de malaria confirmados disminuyó del 6% al 1%, al tiempo que mejoró la detección de los casos. Se registró una disminución de todos los casos de todas las especies de parásitos, incluida la *Plasmodium falciparum*. Aunque el alcance del uso de mosquiteros tratados con insecticidas de efecto prolongado y de mosquiteros impregnados con insecticida, así como la fumigación de interiores con efecto residual por parte de la población en situación de riesgo en las zonas seleccionadas fue elevado, el total de la población no contaba con protección. Además de la detección pasiva, se llevó a cabo la detección activa en las zonas de difícil acceso y en los lugares de trabajo de las poblaciones móviles de inmigrantes. La detección activa mejoró en la mayoría de las zonas entre 2012 y 2014, pero es necesario seguir fortaleciéndola.

Conclusiones: los hallazgos proporcionan datos útiles relativos a la situación de la malaria en las zonas de la iniciativa de la resistencia a la artemisinina, los cuales podrían servir para que el Programa Nacional de Control de la Malaria alcance su meta de eliminación. Estos perfiles podrían contribuir a mejorar la planificación, la ejecución y la evaluación de las actividades de intervención.

Translated from English version into Spanish by MKaszczynice, through

TRANSLATORS
WITHOUT BORDERS
